# Subclinical Mastitis and Antibiotic Exposure Reshape the Milk Bacteriome, Resistome, and Metabolic Functional Profiles in Dairy Cows

**DOI:** 10.1002/mbo3.70440

**Published:** 2026-10-12

**Authors:** M. Nazmul Hoque, M. Shaminur Rahman, Md. Golzar Hossain, Md. Tanvir Rahman, Tofazzal Islam, M. Anwar Hossain

**Affiliations:** ^1^ Molecular Biology and Bioinformatics Laboratory, Department of Gynecology, Obstetrics and Reproductive Health Gazipur Agricultural University Gazipur Bangladesh; ^2^ Jashore University of Science and Technology Jashore Bangladesh; ^3^ Department of Microbiology and Hygiene Bangladesh Agricultural University Mymensingh Bangladesh; ^4^ Institute of Biotechnology and Genetic Engineering Gazipur Agricultural University Gazipur Bangladesh; ^5^ Department of Microbiology University of Dhaka Dhaka Bangladesh

**Keywords:** antibiotic treatment, bovine subclinical mastitis, metabolic pathways, milk bacteriome, resistome

## Abstract

Subclinical mastitis (SCM) reduces milk yield and promotes antibiotic use, contributing to antimicrobial resistance. However, information on milk microbiome dysbiosis and resistome alterations in dairy cows remains limited. We performed shotgun metagenomic sequencing of 30 milk samples from 20 cows classified as SCM (*n* = 10), antibiotic‐treated (ANT; *n* = 10), and healthy milk (HM; *n* = 10). Bacteriome, resistome, and metabolic functions were analyzed using bioinformatic tools. Microbial alpha diversity was significantly higher in HM than SCM and ANT samples (*p* < 0.05), with the greatest richness observed in Local Zebu and Middle‐aged (5–8 years) cows. However, beta diversity did not differ significantly among groups (PERMANOVA, *p* > 0.05). A total of 665 bacterial species belonging to 15 phyla were identified, with Pseudomonadota, Actinomycetota, and Bacillota comprising 90%–99% of total abundance. SCM milk was dominated by *Actinoalloteichus* sp. (40.55%) and *Vibrio furnissii* (37.01%), while *Staphylococcus aureus* was enriched (1.74%) compared with HM and ANT (< 0.01%). ANT milk showed increased abundance of *Acinetobacter johnsonii* (10.52%), whereas HM samples contained beneficial commensals, including *Pseudomonas lundensis*, *Bacteroides thetaiotaomicron*, and *Lactococcus lactis*. Similar taxonomic shifts were observed across breeds and age groups. In total, 194 antimicrobial resistance genes (ARGs) were detected, with SCM harboring the largest repertoire (*n* = 161). Nevertheless, macrolide‐, aminoglycoside‐, and β‐lactam‐resistance genes were significantly enriched in ANT samples (*p* < 0.01). Correlation analyses linked *Escherichia coli* and *A. johnsonii* with multiple ARGs, while *Acinetobacter* and *Pseudomonas* were associated with lipid metabolism, membrane transport, and xenobiotic biodegradation pathways. These findings reveal bacteriome disruption, ARG enrichment, and functional alterations in SCM and antibiotic‐exposed milk.

## Graphical abstract

Overview of the study workflow and metagenomic analysis of bovine milk bacteriomes. Milk samples were collected from dairy cows and classified into three health‐status groups: subclinical mastitis (SCM), antibiotic‐treated (ANT) SCM cows, and healthy milk (HM). Samples were further stratified according to dairy cow breeds (Holstein Friesian Cross; HFC, Local Zebu; LZ, and Sahiwal Cross; SC) and age categories (Young, Middle, and Older). The findings underscore SCM and antibiotic use as key drivers of microbial imbalance and resistome composition, highlighting the need for improved mastitis management strategies.

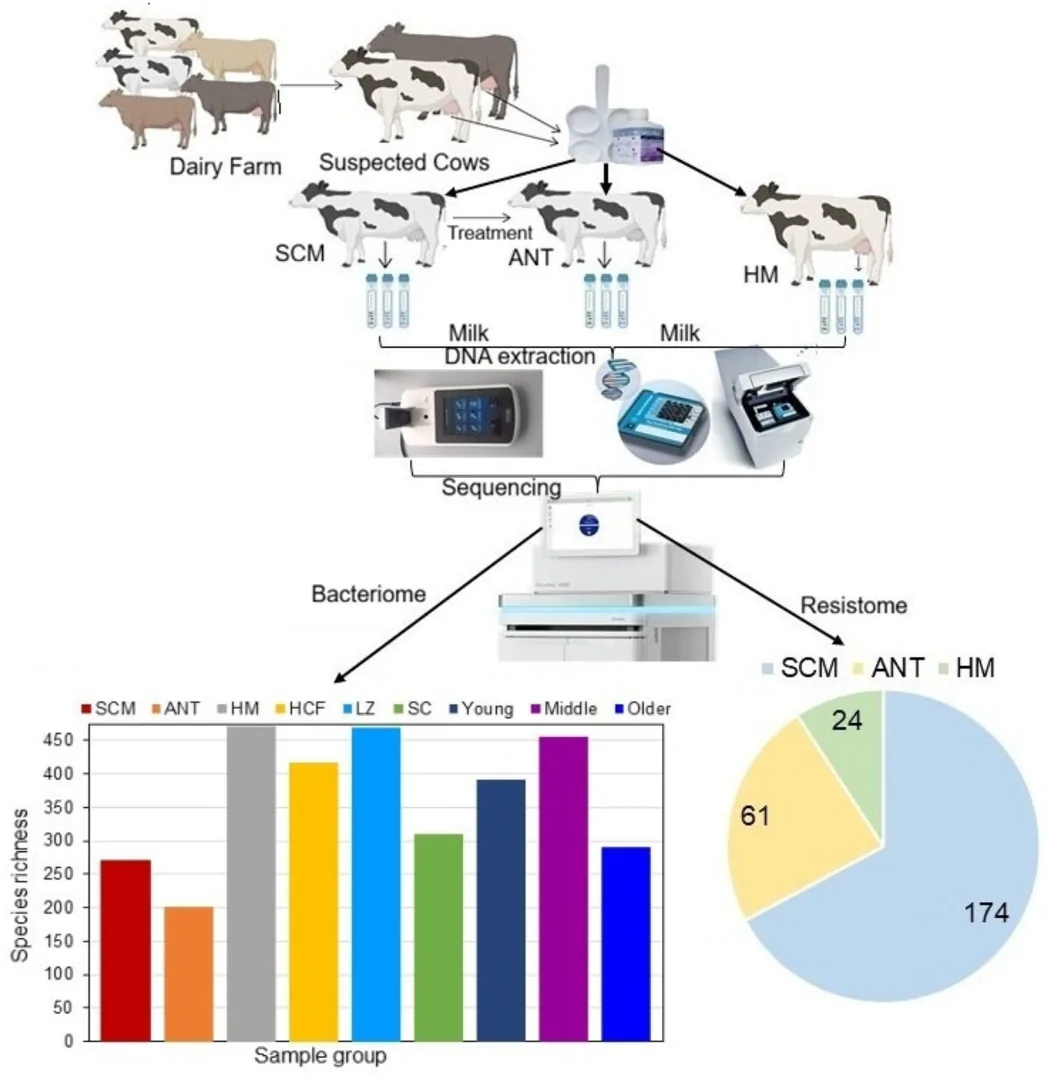

## 1 Introduction

Mastitis, an inflammation of the mammary gland, continues to be one of the most significant challenges in global dairy production. In developing countries like Bangladesh, the burden is particularly severe due to limited veterinary infrastructure, inadequate diagnostic tools, and suboptimal farm management practices (Chowdhury et al. 2025; Hasan et al. 2022). SCM, which is characterized by the absence of visible symptoms, can be detected using tests such as the California Mastitis Test (CMT) (Hoque et al. 2015), and often goes unnoticed by farmers. This silent form of infection reduces milk yield, compromises reproductive efficiency, and necessitates repeated antibiotic interventions, thereby escalating production costs and contributing to the global threat of AMR. Studies across Bangladesh consistently report a high prevalence of SCM (60%–77%), particularly in crossbred and intensively managed cattle (Al Emon et al. 2024; Bhuiyan et al. 2020; Islam et al. 2019), while Singha et al. (2021) highlighted similar issues of SCM in water buffaloes (Singha et al. 2021). These findings emphasize the widespread nature of SCM and its encroachment in both dairy cow and buffalo farming systems in Bangladesh. Compounding the issue is the reliance on broad‐spectrum antibiotics, often administered without culture‐based confirmation of pathogens, which risks promoting resistant strains in both the milk and the broader farm environment (Al Emon et al. 2024; Mahamudul Hasan 2021).

Emerging research highlights the central role of the milk microbiome in bovine udder health (Hoque et al. 2020, 2019; Steinberg et al. 2022). In healthy cows, milk contains a diverse and balanced microbial community, including commensals that may help defend against opportunistic pathogens (Ma et al. 2022). SCM disrupts this equilibrium, leading to dysbiosis characterized by decreased microbial diversity and the overrepresentation of mastitis‐associated bacteria (Derakhshani et al. 2020; Pang et al. 2018). This altered microbiota composition has been implicated not only in the persistence of infection but also in the increased presence of ARGs (Alessandri et al. 2023; Salman et al. 2023a).

Antibiotics are widely used in the dairy industry for treating and preventing mastitis, improving animal health, and maintaining milk production (Hoque et al. 2022b). However, frequent and indiscriminate use contributes to AMR, residue contamination in milk, and disruption of the udder microbiome, raising concerns for public health and food safety (Cheng et al. 2019). Antibiotic treatment intended to manage SCM can further perturb the microbial ecosystem (Cheng et al. 2019; Hoque et al. 2020). Although these therapies aim to eliminate pathogenic bacteria, they may inadvertently suppress beneficial microbial populations, facilitating colonization by resistant organisms (Alessandri et al. 2023; Hoque et al. 2020). Studies in dairy cattle and water buffaloes showed that both SCM and antibiotic treatment are associated with reduced microbial diversity and an enrichment of resistance determinants (Catozzi et al. 2017; Salman et al. 2023b). This proposes that milk from SCM‐affected and antibiotic‐treated cows could act as reservoirs for horizontal gene transfer, exacerbating the AMR problem. Despite these insights, most previous studies on mastitis have relied on culture‐dependent methods that fail to capture the full spectrum of microbial diversity and resistance mechanisms. These methods often overlook non‐culturable or fastidious organisms, and thus offer a limited view of the complex microbial networks within milk (Drago et al. 2017). Additionally, few studies in South Asia have employed high‐resolution metagenomic techniques to investigate the milk microbiome and resistome in a region‐specific context (Hoque et al. 2020, 2019, 2022a). Given the unique climatic, management, and socioeconomic conditions in Bangladesh, there is a pressing need for comprehensive, multi‐omic studies tailored to the local dairy landscape. This study employed shotgun whole metagenome sequencing (WMS) to investigate milk bacteriome dysbiosis, along with corresponding resistome signatures and metabolic functional profiles, in dairy cows across three distinct health conditions—SCM, ANT and HM on a commercial farm in Dhaka, Bangladesh. The findings provide deeper insights into bacterial community dynamics, resistance reservoirs and metabolic functional perturbations, offering valuable implications for improved mastitis management in the dairy industry.

## 2 Materials and Methods

### 2.1 Ethics Statement

All animal procedures were conducted in compliance with standard ethical guidelines approved by the Animal Research Ethics Committee (AREC) of the Gazipur Agricultural University, Bangladesh (Reference number: FVMAS/AREC/2023/6679, Date: 16/01/2023). This manuscript, submitted to MicrobiologyOpen, has been prepared in strict adherence to responsible research practices and the established guidelines of publication ethics.

### 2.2 Sample Collection and Metadata

Thirty milk samples (*N* = 30) were collected from 20 lactating crossbred dairy cows. These samples were represented by three health conditions of the cows *viz*. subclinical mastitis milk (SCM, *n* = 10), antibiotic‐treated milk (ANT, *n* = 10) and healthy milk (HM, *n* = 10) (Table S1). All animals were sourced from a commercial dairy farm in the Dhaka district (23.73° N, 90.38° E) of Bangladesh. SCM was diagnosed based on a positive California Mastitis Test (CMT; CMT Original Schalm, TechniVet, USA) result alone (Hoque et al. 2015). Somatic cell count quantification and bacteriological culture were not performed to confirm diagnosis or bacterial etiology. All 10 CMT‐positive cows were subsequently treated with a 7‐day course of intramuscular injection of ampicillin (β‐lactam; 7 mg/kg body weight) and azithromycin (macrolide; 10 mg/kg body weight), the two most frequently administered antibiotics in dairy animals in Bangladesh. Post‐treatment milk samples were collected 24 h after the final antibiotic injection (Day 8) and classified as ANT milk (*n* = 10) to investigate the effects of SCM and antibiotic exposure on the milk bacteriome, resistome, and metabolic functional profiles of dairy cows. Concurrently, milk samples from 10 healthy, untreated lactating cows were also collected as controls (HM = 10) (**Graphical Abstract**).

The study cohort comprised three predominant breed types: Holstein Friesian Cross (HFC, *n* = 8), Local Zebu (LZ, *n* = 6), and Sahiwal Cross (SC, *n* = 6). Lactation stages ranged from early to mid‐lactation (5–60 days postpartum), and cow ages varied between 4.0 and 10.0 years, categorized into three groups: Young (< 5.0 years), Middle‐aged (5.0–8.0 years), and Older (> 8.0 years) (Table S1). To minimize microbial contamination during sampling, teats were thoroughly cleaned with 70% ethanol and dried using sterile wipes, and the initial milk streams were discarded. Approximately 10 mL of milk was then aseptically collected into sterile tubes, immediately placed on ice (4°C), and transported to the laboratory within 12 h, where samples were stored at –80°C until further processing. Detailed metadata were recorded for each sample, including health status (SCM, ANT and HM), breeds, lactation stage, daily milk yield, and age of the cows. To ensure data accessibility and reproducibility, raw sequencing data have been deposited in the NCBI Sequence Read Archive (SRA) under BioProject accession number PRJNA1081607, with individual sample accessions provided in Table S1. This comprehensive metadata collection enables robust analysis of potential confounding factors, including breed‐specific differences, lactation physiology, and regional farming practices.

### 2.3 DNA Extraction and Sequencing

Genomic DNA was extracted from 30 milk samples, comprising equal subsets of SCM (*n* = 10), ANT (*n* = 10), and HM (*n* = 10) specimens, using an automated platform following established protocols (Hoque et al. 2019, 2020). DNA quantity and purity were determined using a NanoDrop ND‐2000 spectrophotometer (ThermoFisher, USA) by measuring the 260/280 absorbance ratio. Shotgun WMS libraries were prepared using the Illumina Nextera XT DNA Library Preparation Kit according to manufacturer specifications. Paired‐end sequencing (2 × 150 bp) was performed on the NovaSeq 6000 platform (Illumina Inc., USA), generating a total of 327.0 million raw reads across all samples. The data set demonstrated robust coverage, with individual samples yielding an average of 10.90 million reads (range: 3.30–19.48 million), ensuring sufficient depth for comprehensive metagenomic analysis (Table S1). All DNA extractions and sequencing library preparations were performed using negative extraction controls (sterile nuclease‐free water processed alongside samples) to monitor for potential reagent or environmental contamination. Any putative microbial or ARG signals detected in these negative controls were rigorously subtracted from the sample data sets during bioinformatic analysis.

### 2.4 Bioinformatics Analysis

Raw sequencing data underwent rigorous quality control and processing through a standardized bioinformatics pipeline. Initial read quality assessment was performed using FastQC (v0.11.6) (Andrews 2010). Adapter trimming and quality filtering were performed using Trimmomatic v0.39 (Bolger et al. 2014) with a 4‐bp sliding window, a minimum Phred quality score of 20, and a minimum read length of 50 bp. This process yielded 318.62 million high‐quality reads, averaging 10.62 million read pairs per sample (range: 3.25–19.27 million) (Table S1). To separate microbial sequences, host‐derived reads were removed in silico using BBduk (BBtools package v38.x) (Bushnell 2014). This was achieved by aligning the quality‐trimmed reads to the *Bos taurus* reference genome (GCF_002263795.1) with a k‐mer length of 31; reads that did not align were classified as “host‐filtered” and used for all subsequent analyses. Host read filtering effectively removed a small proportion of host‐derived sequences across all samples, with the majority of reads (94%–99%) retained as unmapped for downstream microbial analysis. Taxonomic profiling was performed on the host‐filtered reads using Kraken2 (v2.1.x) with a custom database (latest update July 2025). The classification was run with a confidence threshold of 0.1 and a requirement for a minimum of 2 hit groups. To refine the abundance estimates at the species level, the Kraken2 (Wood et al. 2019) output was processed with Bracken (Bayesian Reestimation of Abundance after Classification with KrakEN) (Lu et al. 2017). Finally, taxonomic identifiers were mapped to their full scientific nomenclature using TaxonKit (Shen and Ren 2021).

Functional profiling of resistance determinants (i.e., resistome) and metabolic pathways was performed using a comprehensive bioinformatics workflow. For resistome analysis, high‐quality metagenomic reads were processed through the AMR + + pipeline and aligned against the MEGARes v2.0 database (Bonin et al. 2023) using the Burrows–Wheeler Aligner (Li and Durbin 2010). To ensure high‐confidence ARG identification, only alignments covering ≥ 99% of the reference gene length were retained. The filtered ARG profiles were subsequently analyzed with ResistomeAnalyzer v2.1 (https://github.com/cdeanj/resistomeanalyzer) to characterize AMR patterns, thereby minimizing false‐positive detections arising from partial gene matches. Metabolic functional profiling was conducted using HUMAnN3 (Murovec et al. 2024)‐derived Reads Per Kilobase (RPK) gene family abundance tables. Duplicate UniRef90 entries were consolidated by summing their abundances after removal of taxonomic annotations. UniRef90 identifiers (Murovec et al. 2024) were then mapped to KEGG Orthology (KO) terms using the *map_ko_uniref90.txt* reference file. Where a gene corresponded to multiple KOs, its abundance was proportionally distributed among the associated orthologs (Kanehisa et al. 2023). Sample‐specific KO abundance tables were generated and merged into a unified matrix, with missing values replaced by zeros. Functional annotation and pathway reconstruction were performed using the KEGGREST R package (Tenenbaum et al. 2019), enabling pathway‐level abundance estimation and comparative analysis of microbial metabolic functions across samples. This analytical framework provided detailed metabolic insights while ensuring compatibility with subsequent statistical and comparative analyses.

### 2.5 Statistical Analysis

Microbial diversity analyses were conducted in R (v4.2.0) using RStudio (2022.07.1), primarily leveraging the phyloseq (v1.50.0) (McMurdie and Holmes 2013) and vegan (v2.6‐4) (Oksanen et al. 2013) packages. To assess differences in alpha diversity between groups, appropriate non‐parametric tests were applied according to the study design: Wilcoxon signed‐rank tests were used for the paired comparison between SCM and ANT samples (pre‐ and post‐treatment), while standard Wilcoxon rank‐sum tests were applied for independent two‐group comparisons involving the healthy control (HM) group. For multi‐group comparisons across all three sample types (SCM, ANT, HM), Kruskal–Wallis tests followed by Dunn's post hoc tests with Bonferroni correction were performed. Beta diversity was assessed using Bray–Curtis dissimilarity matrices derived from TSS‐normalized counts. Differences in community composition among sample type (SCM, ANT, HM), breed type (HFC, SC, and LZ), and age group (Young, Middle‐Aged, and Older) were tested by permutational multivariate analysis of variance (PERMANOVA) implemented in “adonis2” (vegan v2.6‐4) (Oksanen et al. 2013), with 999 permutations. Because SCM and ANT samples were collected from the same 10 cows before and after antibiotic treatment, whereas HM samples were obtained from 10 different untreated cows, the study follows a mixed design with a repeated‐measures (paired) component. PERMANOVA was therefore applied in a design‐specific manner. For the paired SCM versus ANT comparison, permutations were restricted within individual cows (cow ID as blocking factor, specified through the “strata” argument of adonis2), so that group labels were exchanged only between the pre‐ and post‐treatment samples of the same animal. For comparisons of HM with SCM and with ANT, which involve different animals, unrestricted (free) permutations were used. The overall three‐group PERMANOVA (SCM, ANT and HM) was run with unrestricted permutations and is reported descriptively, because a single permutation scheme cannot simultaneously respect the within‐cow pairing and the between‐cow structure of the design; inference on health‐status effects was therefore based on the design‐appropriate pairwise tests. Because breed and age are constant within each cow, PERMANOVA for these variables was performed on the 20 independent animals only (one sample per cow, i.e., the pre‐treatment SCM sample and the HM sample), with health status included as a covariate, so that repeated samples from the same cow were not treated as independent observations. Effect sizes were reported as *R* ^2^ values, and ordination was visualized by principal coordinates analysis (PCoA) using the ordinate function in “phyloseq”. All *p*‐values were adjusted for multiple testing using the Benjamini–Hochberg false discovery rate, with *p* < 0.05 considered statistically significant. Spearman's rank correlation was used to assess associations among the top 50 taxa based on relative abundance. Strong correlations (|*ρ* | > 0.5) were visualized using chord diagrams generated with the “circlize” package (Gu et al. 2014) in R. Additional Spearman's correlation analyses were performed to evaluate relationships among dominant bacterial species, ARGs, and KEGG functional pathways. The R packages, “hmisc” (Harrell and Harrell 2019) and “corrplot” (Wei et al. 2017), were used respectively to analyze and visualize the data.

## 3 Results

### 3.1 Microbial Diversity Across Sample Groups

Microbial alpha diversity varied significantly across sample types, breed types and age groups (Kruskal–Wallis test, *p* < 0.05) (Figure 1). Healthy milk (HM) samples exhibited the highest microbial richness (median = 129.5 observed taxa) and Shannon diversity (median = 1.59), compared to SCM and ANT samples, which showed reduced diversity (SCM: median richness = 68.5, Shannon = 1.14; ANT: median richness = 51.5, Shannon = 1.41). Pairwise comparisons confirmed significantly higher diversity in HM relative to both SCM (*p* = 0.03) and ANT (*p* = 0.01) (Figure 1A). Breed type also influenced alpha diversity, with LZ samples showing the highest observed richness (median = 142.0) and Shannon diversity (median = 1.78), followed by HFC (median richness = 101.0, Shannon = 1.45) and SC (median richness = 81.0, Shannon = 1.21). Significant differences were observed between LZ and SC (*p* < 0.01) and between HFC and SC (*p* < 0.05) (Figure 1B). Age‐related trends showed that Middle‐aged animals (5.0–8.0 years) exhibited peak diversity (median richness = 132.0, Shannon = 1.68), significantly higher than Young (< 5 years; median richness = 84.5, Shannon = 1.24; *p* < 0.01) and Older (> 8.0 years; median richness = 91.0, Shannon = 1.36; *p* < 0.05) groups (Figure 1C). A weak negative correlation was observed between lactation stage and Shannon diversity (*ρ* = −0.32, *p* = 0.08), while milk yield showed no significant association with microbial diversity (*ρ* = 0.15, *p* = 0.41).

**Figure 1:**
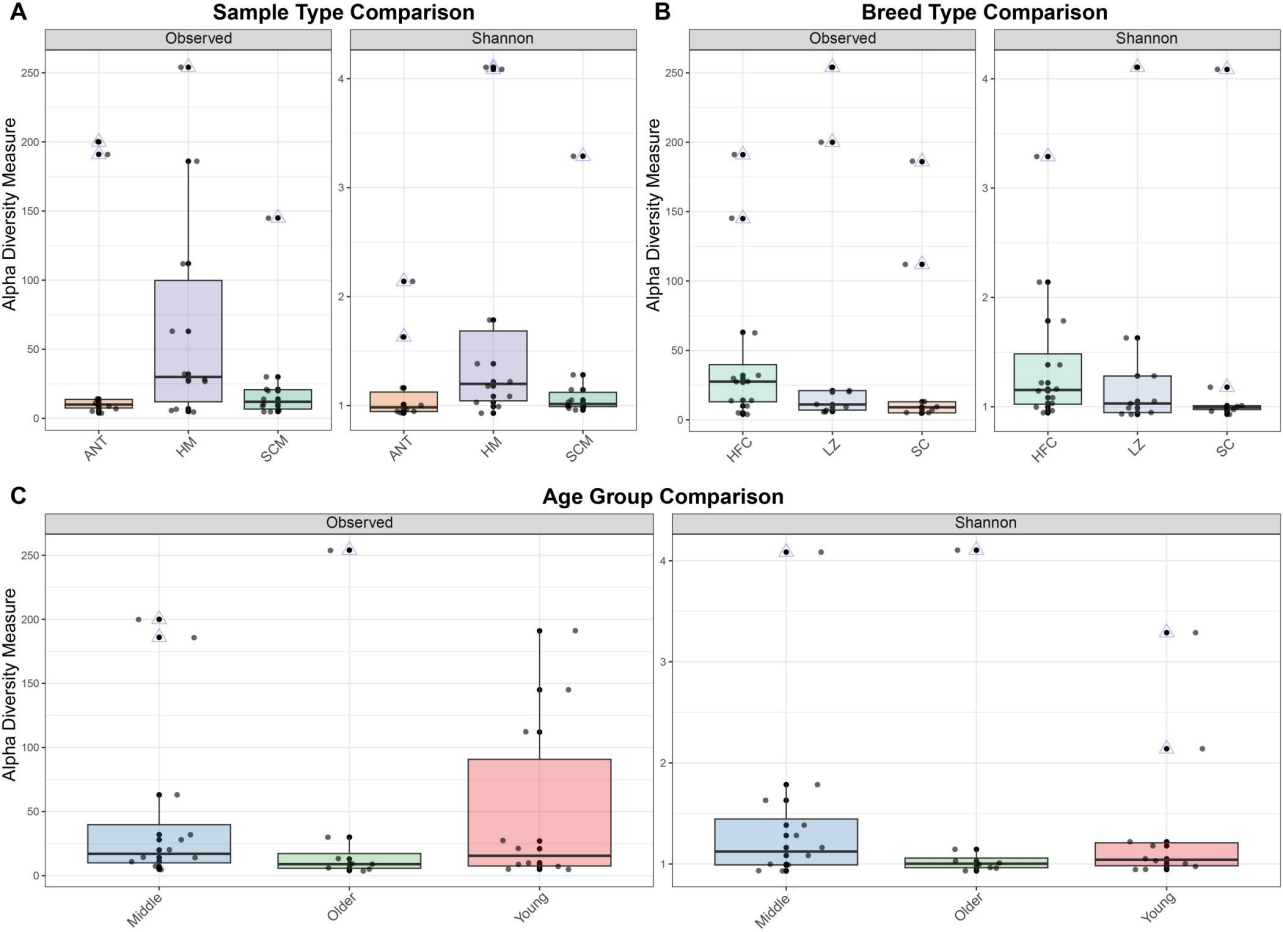
Alpha diversity of the microbial communities. Boxplots showing Observed richness and Shannon diversity indices for (A) Sample Type (SCM = Subclinical Mastitis Milk, ANT = Antibiotic Treated Milk, HM = Healthy Milk), (B) Breed Type (HFC = Holstein Friesian Cross, SC = Sahiwal Cross, LZ = Local Zebu), and (C) Age group (Young = < 5 years, Middle‐aged = 5.0–8.0 years, Older = > 8.0 years). Each panel displays the distribution of alpha diversity metrics with individual samples shown as jittered points. Boxplots represent the median (horizontal line), interquartile range (box), and whiskers extending to 1.5× the interquartile range. Outliers are shown as blue triangles. Statistical comparisons between groups were performed using Wilcoxon signed‐rank and Wilcoxon rank‐sum tests, with only significant pairwise differences (*p* < 0.05) indicated by asterisks (*p < 0.05, **p < 0.01, ***p < 0.001, ****p < 0.0001). Non‐significant comparisons are not shown to reduce visual clutter.

Beta diversity was evaluated using PCoA based on Bray‐Curtis dissimilarities, and statistical significance was tested using PERMANOVA (adonis2). As shown in Figure 2, no statistically significant differences in microbial community composition were observed across the tested metadata categories (PERMANOVA, *p* > 0.05). Sample type accounted for 9.34% of the microbial community variance (overall three‐group PERMANOVA with unrestricted permutations: *R* ^2^ = 0.0934); however, the separation among ANT, HM, and SCM groups was not significant (*p* = 0.142). Breed type explained 8.72% of the variation (*R* ^2^ = 0.0872, *p* = 0.810) (Figure 2A). Age group explained 7.68% of the variation (*R* ^2^ = 0.0768, *p* = 0.831) (Figure 2B). For age group comparisons, Middle‐aged samples formed a distinct cluster partially overlapping with young and older groups, consistent with alpha diversity patterns showing peak diversity in Middle‐aged animals (Figure 2B). While all tested variables showed no statistically significant effects, the relatively low *R* ^2^ values (< 1.0) indicate that the majority of microbial variation remains unexplained, suggesting that other factors beyond health status may play a more substantial role in shaping the milk microbiota.

**Figure 2:**
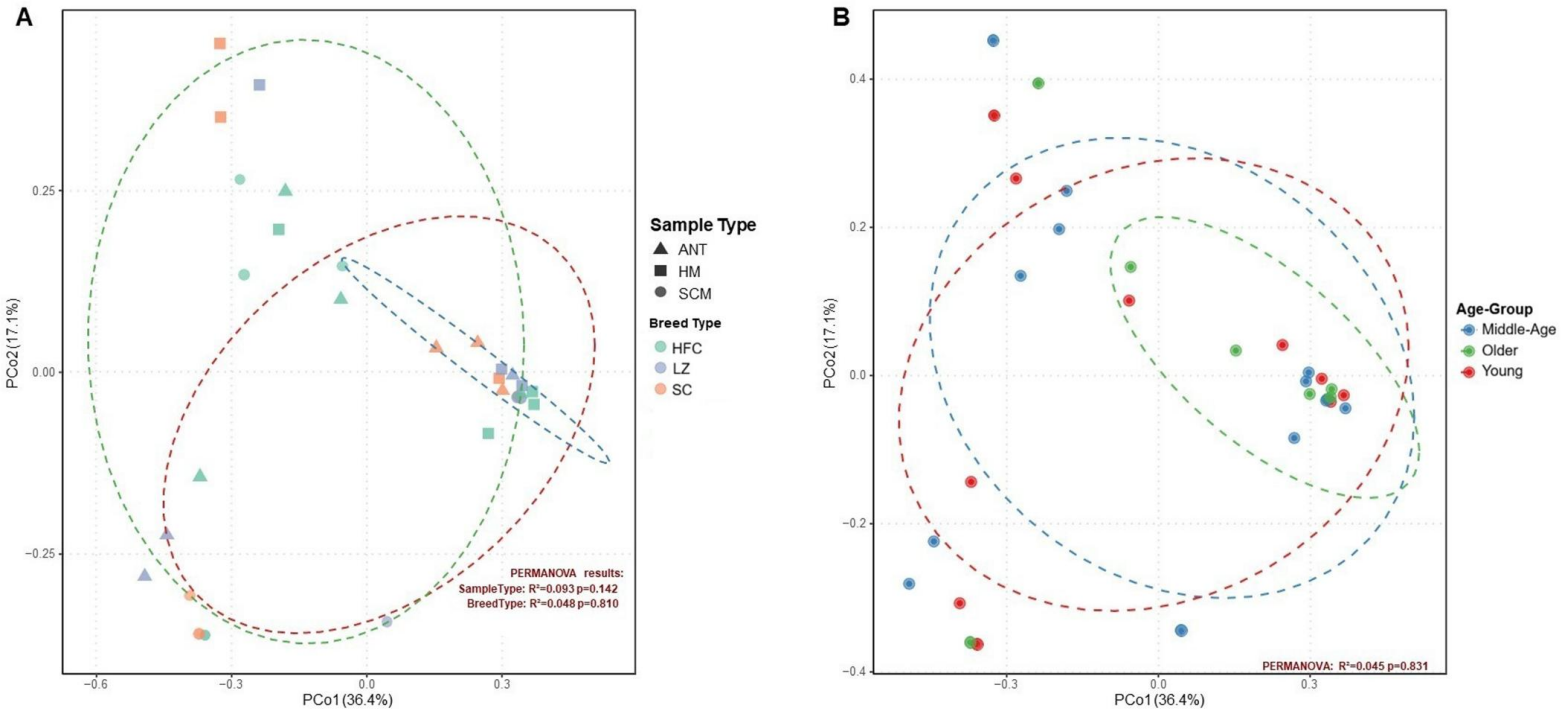
Beta diversity of the microbial communities based on Bray‐Curtis distances. Principal Coordinates Analysis (PCoA) ordination plots showing microbial community composition across different metadata categories. (A) PCoA plot displaying samples colored by Sample type (SCM, ANT and HM) and Breed type (HFC, LZ and SC). The percentage of variance explained by each principal coordinate is shown in axis labels. (B) PCoA plot focusing on Age group (Young = < 5 years, Middle‐aged = 5.0–8.0 years, Older = > 8.0 years) comparisons, with samples colored by age category, and dashed ellipses representing 68% confidence intervals for each group. Statistical significance of community composition differences was assessed using PERMANOVA (permutational multivariate analysis of variance) with 999 permutations. Results are displayed as annotations: *R* ^2^ values indicate the proportion of variance explained by each grouping variable, and *p*‐values indicate statistical significance (*p* < 0.05 considered significant). Design‐specific PERMANOVA models were fitted with both restricted and unrestricted permutations of all 30 samples and therefore do not incorporate the pairing of the SCM and ANT samples within the same 10 cows (see Section 2.5).

### 3.2 Phylum‐Level Bacteriome Composition and Structure Across Study Groups

At the domain level, bacteria were the most abundant community, with an average abundance of 99.57%, followed by archaea (0.32%), and viruses (0.11%). At the phylum level, the bacteriome comprised 15 phyla, with significant differences in microbial community composition observed across sample type, age, and breed groups (Kruskal–Wallis test, *p* < 0.03) (Figure 3 and Figure S1). Among sample types, only four phyla (26.67%) were shared across ANT, HM, and SCM samples, with SCM harboring the highest number of phyla (*n* = 11), followed by HM (*n* = 10) and ANT (*n* = 7) (Figure S1A). Among breed groups, seven phyla (46.67%) were shared among breed groups, with HFC and LZ harboring 11 phyla each and SC containing 10 phyla (Figure S1B). Similarly, seven phyla (46.67%) were common to the Young, Middle‐Aged, and Older groups, with 11 phyla detected in both Young and Older animals and nine in the Middle‐aged group (Figure S1C). Across all comparisons, Pseudomonadota, Actinomycetota, and Bacillota were the dominant phyla, collectively accounting for 90.00%–99.00% of the total bacterial abundance (Figure 3 and Data S1). In sample type groups, Pseudomonadota was more abundant in ANT (51.75%) and HM (40.57%) than in SCM (37.80%), whereas Actinomycetota was enriched in SCM (40.67%) and HM (32.80%), and Bacillota was highest in HM (16.19%), followed by SCM (14.56%) and ANT (8.70%) (Figure 3A and Data S1). Among breeds, Pseudomonadota was most abundant in SC (44.37%), followed by HFC (43.26%) and LZ (42.52%), whereas Actinomycetota showed comparable abundances in HFC (38.84%) and LZ (38.10%) but lower abundance in SC (35.65%). Bacillota was enriched in SC (14.94%) and LZ (13.94%) compared with HFC (11.22%) (Figure 3B and Data S1). Across age groups, Pseudomonadota declined from Young (48.49%) to Middle‐aged (42.41%) and Older (38.41%) animals, while Actinomycetota increased from Young (33.71%) to Middle‐aged (38.92%) and Older (40.71%) groups. Bacillota was more abundant in Older (14.76%) and Middle‐aged (14.03%) animals than in Young animals (10.80%) (Figure 3C, and Data S1). Other phyla, including Bacteroidota, Spirochaetota, and Methanobacteriota, occurred at low relative abundances (< 7%), while several rare phyla were detected only in specific health, age, or breed groups, indicating niche‐specific microbial specialization.

**Figure 3:**
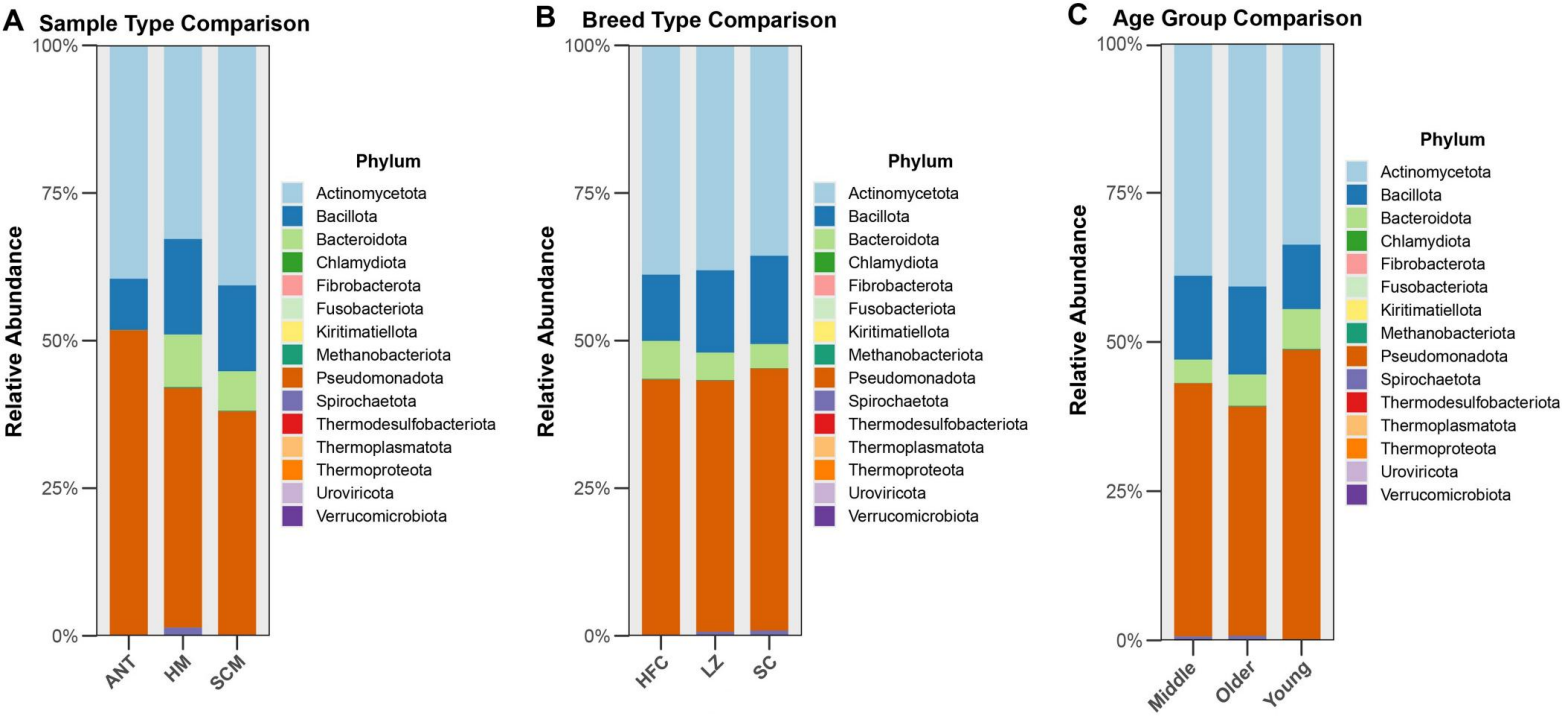
Relative distribution of bacterial phyla across the sample groups. Stacked bar charts display the composition and relative abundance of bacterial communities at phylum level according to (A) sample types (SCM, ANT, and HM), (B) breeds (HFC, LZ, and SC), and (C) age (Young, Middle, Older) of the dairy cows. The *X*‐axis indicates the sample groups, while the *Y*‐axis represents the percentage of relative abundance. Each color corresponds to a distinct bacterial phylum, enabling visual comparison of microbiome profiles across groups.

### 3.3 Species‐Level Bacteriome Composition and Structure Across Study Groups

A total of 665 bacterial species were identified including 471 in HM group followed by 272 in ANT and 202 in SCM metagenomes, of which only 26 (3.91%) species were present in the three sample sets (Figure S2A). Species‐level assessment revealed notable differences in microbial profiles among the ANT, HM, and SCM groups (Figure 4A and Data S1). Kruskal–Wallis tests identified several taxa with significantly different abundances across groups (*p* < 0.05). *Actinoalloteichus* sp. was highly abundant in all three groups, with the highest prevalence in SCM (40.55%), followed by ANT (39.51%) and HM (31.11%). *Vibrio furnissii* was also abundant across groups, peaking in SCM (37.01%), compared to ANT (32.13%) and HM (27.15%). *Acinetobacter johnsonii* was most abundant in ANT (10.52%), moderately present in HM (2.50%), and almost absent in SCM (< 0.01%), indicating potential inhibition or competitive exclusion in the SCM environment. The relative abundance of *A. johnsonii* differed markedly among groups, being highest in the ANT group (10.52%), intermediate in the HM group (2.50%), and nearly absent in the SCM group (< 0.01%), suggesting that the subclinical mastitis microenvironment may be unfavorable for colonization or survival of this species. *Bacillus amyloliquefaciens* showed comparable levels in both SCM (9.79%) and ANT (8.62%), with slightly lower abundance in HM (7.21%). *Bacteroides thetaiotaomicron*, a commensal gut microbe, was detected in HM (2.64%) and SCM (3.68%) but not detected in ANT samples. The relative abundance of *S. aureus*, a key mastitis‐associated pathogen, was markedly higher in the SCM group (1.74%) than in the HM and ANT groups (< 0.01%), underscoring its close association with the dysbiotic microbial environment characteristic of SCM*. Pseudomonas lundensis* was detected in both the ANT (1.58%) and HM (6.41%) groups but was completely absent from SCM samples. Similarly, *Pseudomonas helleri* was identified in the ANT (1.67%) and HM (0.60%) groups, while it remained undetectable in the SCM group (Figure 4A and Data S1). The absence of these species in mastitic milk indicates a potential negative association with subclinical mastitis and suggests that these taxa may be displaced during disease‐associated microbial dysbiosis. Wilcoxon pairwise comparisons revealed that certain opportunistic pathogens tended to be more abundant in SCM and ANT groups, though these differences did not always reach statistical significance after multiple testing correction. Overall, these findings suggest that while some potentially pathogenic taxa are enriched in SCM, commensal species such as *B. thetaiotaomicron* and *P. lundensis* were more abundant in HM, highlighting subtle but species‐specific compositional differences among the three groups.

**Figure 4:**
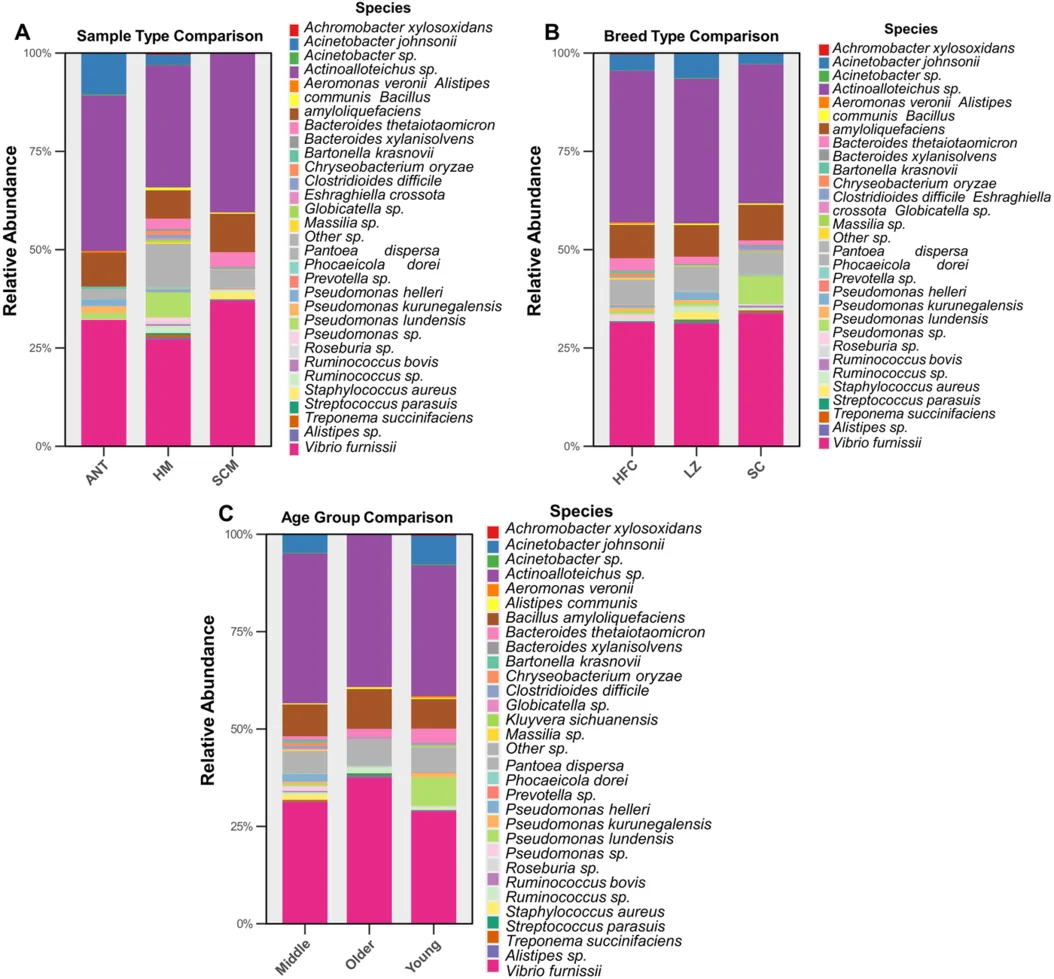
Relative distribution of the top abundant 30 bacterial species across the sample groups. (A) Stacked bar charts display the composition and relative abundance of bacterial species according to sample types (SCM, ANT, and HM), (B) breeds (HFC, LZ, and SC), and (C) age (Young, Middle, Older) of the dairy cows. The *X*‐axis indicates the sample groups, while the *Y*‐axis represents the percentage of relative abundance. Each color corresponds to a distinct bacterial species, enabling visual comparison of microbiome profiles across groups.

Analysis of bacterial species abundances across the three breed types (HFC, LZ, and SC) revealed both shared and breed‐specific patterns. In these breeds, the highest number of bacterial species were detected in LZ (*n* = 470) followed by HFC (*n* = 416), and SC (*n* = 310). Among these species, 29.02% were shared across HFC, LZ, and SC (Figure S2B). In all breed groups, *Actinoalloteichus* sp. was the most abundant species, with the highest relative abundance in HFC (38.54%), followed by LZ (36.75%) and SC (35.39%). *V. furnissii* was also highly abundant across all breeds, showing the highest abundance in SC (33.79%), compared to HFC (31.52%) and LZ (31.16%). *B. amyloliquefaciens* was consistently present, with abundances of 8.52% in HFC, 8.06% in LZ, and 9.04% in SC (Figure 4B and Data S1). Notably, *A. johnsonii* was more abundant in LZ (6.35%) compared to HFC (4.03%) and SC (2.75%). *P. lundensis* showed striking breed‐specific enrichment in SC (7.12%), whereas its abundance remained below 0.80% in both HFC and LZ. *B. thetaiotaomicron* was most abundant in HFC (3.06%), followed by LZ (1.85%) and SC (1.08%). *Ruminococcus* sp. peaked in LZ (1.32%), compared to 0.58% in HFC and 0.70% in SC. The rest of the bacterial species exhibited lower relative abundances (< 2.0 in all groups), and showed breed‐specific variations (Figure 4B and Data S1). These quantitative differences at the species level support the previously observed phylum‐level trends, particularly the enrichment of *Pseudomonadota* in SC and *Actinomycetota* in HFC and LZ.

Analysis of bacterial species abundance across the three age groups (Young, Middle, and Older) revealed clear age‐associated shifts in milk microbiota composition. The Middle‐aged group harbored the greatest species richness (*n* = 455), followed by the Young (*n* = 391) and Older (*n* = 291) groups, with only 20.30% of species shared among all three age categories (Figure S2C). *Actinoalloteichus* sp. remained the dominant species across all groups, increasing progressively from 33.63% in Young cows to 38.48% in Middle‐aged and 39.20% in Older cows. Similar age‐related increases were observed for *V. furnissii* (28.84%, 31.25%, and 37.44%, respectively) and *B. amyloliquefaciens* (7.61%, 8.18%, and 10.24%, respectively) (Figure 4C, and Data S1). In contrast, *P. lundensis* was most abundant in Young animals (7.36%), declined sharply in Middle (0.52%), and was not detected in the Older group. *A. johnsonii* followed a similar pattern, with the highest abundance in Young (7.28%), moderate levels in Middle (4.79%), and < 0.01% in the Older animals. *B. thetaiotaomicron* decreased from 3.68% in Young to 0.81% in Middle, but remained elevated in Older (2.08%). *Ruminococcus* sp. showed a different trend, decreasing from 0.70% in Young to 0.52% in Middle (slight decrease) and then rising to 1.48% in Older cows. Several species, including *P. helleri* (1.88%), *Staphylococcus aureus* (1.37%), *Chryseobacterium oryzae* (0.80%), and *Clostridioides difficile* (0.63%), exhibited peak abundance in the Middle‐aged group. Conversely, *Alistipes communis*, *Phocaeicola dorei*, *Treponema succinifaciens*, *Roseburia* sp., and *Corynebacterium xerosis* displayed progressive enrichment with age, reaching their highest abundances in Older cows (0.21–0.48%). In contrast, *Pantoea dispersa*, *Kluyvera sichuanensis*, *Klebsiella pneumoniae*, and *Aeromonas veronii* were relatively enriched in Young cows (0.36–0.42%) and decreased steadily with advancing age (Figure 4C and Data S1).

### 3.4 Bacterial Community Structure and Ecological Relationships

The present study revealed a highly skewed bacteriome composition in bovine milk samples, with a small number of bacterial species dominating the ecosystem (Figure 5 and Data S1). *Actinoalloteichus* sp. and *V. furnissii* emerged as the two most abundant species in the ANT sample group (39.5% and 32.1%, respectively), together accounting for over 70% of the total community. *A. johnsonii* (10.5%) ranked as the third most abundant species, followed by *B. amyloliquefaciens* (8.6%). These four taxa collectively dominated the bacteriome, with all remaining species occurring at abundances below 2% (Data S1). The microbial network analysis revealed ecologically relevant co‐occurrence patterns among the detected species (Figure 5). Notably, all pairwise Spearman correlations in the data set were positive, indicating predominantly cooperative or co‐occurring rather than competitive relationships among these taxa. *V. furnissii* exhibited a strong positive correlation with *B. amyloliquefaciens* (*r* = 0.93), representing one of the most prominent associations among the dominant species. *A. johnsonii* demonstrated positive associations with multiple *Pseudomonas* species, including *P. helleri* (*r* = 0.79), *P. kurunegalensis* (*r* = 0.70), *P. lundensis* (*r* = 0.70), and *Pseudomonas* sp. (*r* = 0.63). Similarly, *P. lundensis* showed strong positive correlations with *P. kurunegalensis* (*r* = 1.00) and *P. helleri* (*r* = 0.83), as well as with *A. veronii* (*r *= 0.77) and *P. dispersa* (*r* = 0.77). Besides, *P. dispersa* and *A. veronii* displayed the strongest inter‐genus association in the data set (*r* = 1.00), representing a near‐perfect co‐occurrence pattern. *P. helleri* similarly co‐occurred with *P. kurunegalensis* (*r* = 0.84) and *Pseudomonas* sp. (*r* = 0.84), underscoring the tight ecological coupling within the *Pseudomonas* cluster (Figure 5 and Data S1). A distinct commensal consortium was identified among gut‐associated bacterial genera, including *B. thetaiotaomicron*, *B. xylanisolvens*, *A. communis*, *Alistipes* sp., *C. difficile*, *Eshraghiella crossota*, *Globicatella* sp. PHS‐GS‐PNBC‐21‐1553, *Prevotella* sp., *Roseburia* sp., *Ruminococcus bovis*, *Ruminococcus* sp., and *T. succinifaciens*, all of which displayed remarkably strong mutual co‐occurrence (*r *= 0.99–1.00). *B. thetaiotaomicron* and *B. xylanisolvens* showed a perfect correlation (*r* = 1.00), and *Streptococcus parasuis* positively correlated with all members of this consortium (*r* = 0.80), while *P. dorei* also participated in this network (*r* = 0.88 with most consortium members). This tightly interconnected cluster likely represents a conserved commensal assemblage co‐detected across samples (Figure 5 and Data S1).

**Figure 5:**
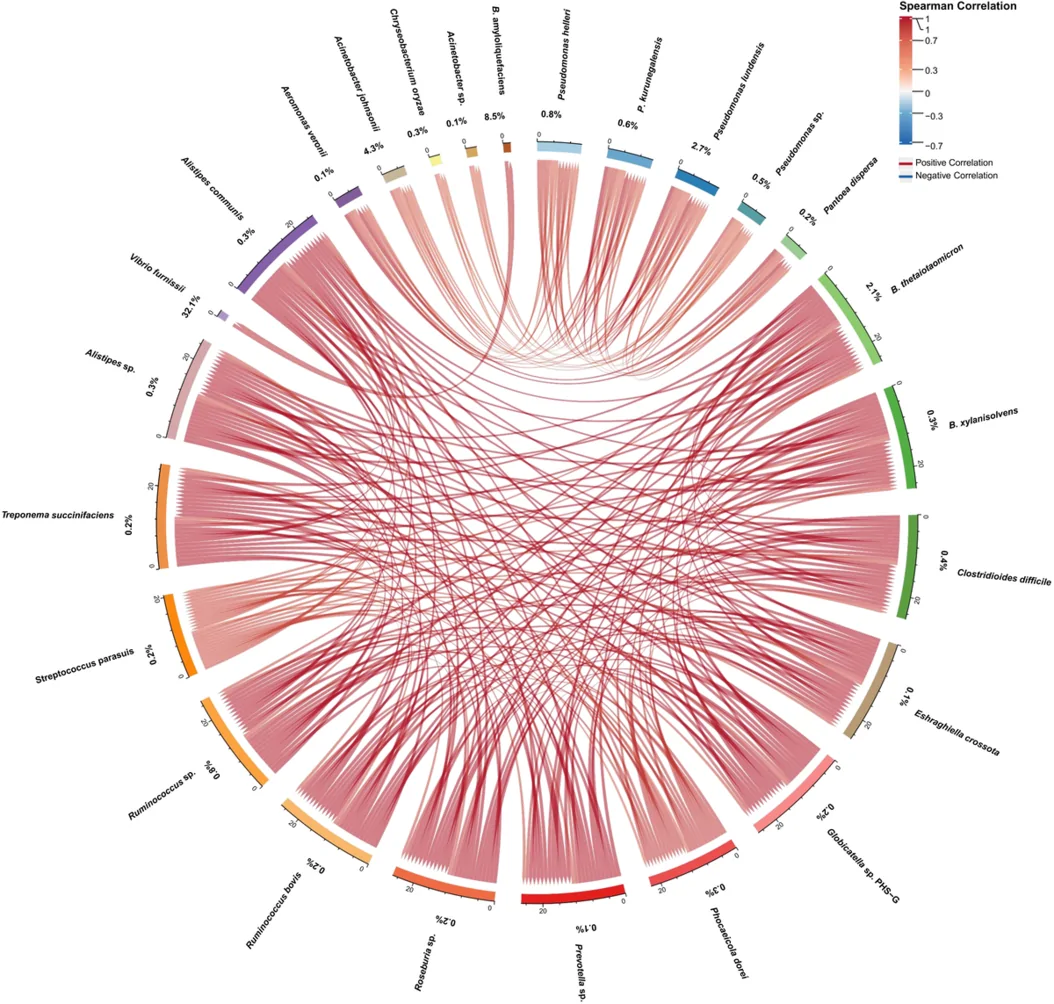
Microbial interactions among the top 25 dominant species. A chord diagram was constructed to visualize Spearman correlation‐based interactions among the 25 most abundant microbial species identified across the study groups. Only significant correlations (|*ρ* | > 0.5) were included in the network. The diagram displays positive correlations in orange and negative correlations in moss green, with the width and intensity of each chord representing the strength of association. A color gradient and detailed legend were incorporated to enhance interpretability.

### 3.5 Resistome Profile Across Sample Groups

The comparative analysis of ARGs demonstrated clear variation in resistome profiles across SCM, ANT, and HM groups (Data S1). Overall, 194 distinct ARGs were identified, with the majority detected in SCM‐associated microbiomes (*n* = 161), followed by ANT (*n* = 51) and HM (*n* = 24) (Figure S3). Among these ARGs, 11 (5.67%) were found to be shared across all three groups, indicating a highly divergent resistome structure. SCM microbiomes possessed the largest fraction of unique ARGs (68.04%), highlighting their role as a major reservoir of resistance determinants, whereas ANT and HM microbiomes accounted for 11.86% and 4.12% of unique ARGs, respectively (Figure S3). Macrolide resistance genes, particularly the 23S rRNA mutation (*MLS23S*), dominated in ANT samples (15.7%–21.9%), reflecting strong selection pressure from clinical macrolide use. Aminoglycoside resistance mechanisms, such as 16S ribosomal mutations (*A16S*; 10.7%–11.9%) and protective ribosomal proteins (RRSH; 8.5%–9.0%), were similarly enriched in ANT samples, confirming treatment‐associated resistance development in mastitis pathogens. Notably, β‐lactamase genes showed differential distribution, with class D enzymes (*OXA*) prevalent in ANT milk (1.4%) while class A (*CFX*) appeared in both SCM (2.4%) and HM (1.8%–1.9%) samples. The ARG profiles revealed clinically important patterns, with multidrug efflux pump‐related ARG (*e.g., TTGB*; 3.6%–3.8%) significantly higher in ANT milk samples, while biocide/metal resistance gene (*RUVB*) appeared in both SCM (2.4%) and HM (1.15%) samples. The HM samples also harbored resistance genes related to tetracycline ribosomal protection proteins (TETQ; 3.0%) and elfamycin resistance factors (TUFAB; 3.4%). Statistical comparisons revealed that milk samples from antibiotic‐treated animals (ANT) contained significantly higher levels of resistance genes against macrolides, lincosamides, streptogramins (MLS), and β‐lactams (*p* < 0.05). In contrast, SCM samples were characterized by an enrichment of efflux pump genes (*ACRB*) and metal resistance genes (*ZNTA*), which significantly differentiated them from HM samples (*p* < 0.01). The strong positive correlation between macrolide and aminoglycoside resistance (*r* = 0.72) in ANT milk indicates co‐selection of resistance mechanisms under therapeutic pressure, whereas negative associations between HM‐associated efflux pumps and concurrent resistance genes (*TTGB*, *TTGC*; *r* = −0.65) may reflect competing resistance strategies in treated versus untreated animals (Data S1).

### 3.6 Correlation Between ARGs and Top Abundant Bacterial Species

Spearman correlation analysis identified several statistically significant associations between ARGs and abundant bacterial taxa (*p* < 0.05) (Figure 6). These results are based on correlation patterns derived from compositional metagenomic data and should not be interpreted as evidence of direct ecological or mechanistic interactions. Strong positive associations were observed for *E. coli* with multiple ARGs, including tetracycline resistance genes (*TET32*, *TETQ*, *TETM*; *r* = 0.63), aminoglycoside resistance genes (*ANT3*‐*DPRIME*; *r *= 0.63), and efflux pump–related genes (*TTGB*, *TTGC*; *r* = 0.63). *Actinoalloteichus* sp. and *V. furnissii* showed broad positive correlations across nearly all ARG categories, including β‐lactam (*CFX*; *r* = 0.36), *MLS* (*MEFA*, *MEEE*, *MEXE*; *r* ≈ 0.31–0.34), and tetracycline resistance genes (*TET32*, *TETQ*; *r *≈ 0.31–0.34). *A. johnsonii* was positively correlated with aminoglycoside resistance genes (*A16S*; *r* = 0.79) and β‐lactam resistance genes (*CFX*; *r* = 0.66). *K. pneumoniae* showed positive correlations with efflux pump–associated genes (*TTGB*, *TTGC*; *r* = 0.69) and tetracycline resistance genes (*TETM*; *r* = 0.69). *Massilia* sp. exhibited consistent modest negative correlations across nearly all ARGs (*r* ≈ −0.13 to −0.18), as did *Bartonella krasnovii* (*r* ≈ −0.15 to −0.17). *P. lundensis* showed modest positive correlations with β‐lactamase genes (*OXA*; *r* = 0.10) and aminoglycoside genes (*A16S*; *r* = 0.09) (Figure 6). Overall, these associations describe co‐occurrence patterns between ARGs and bacterial taxa in the milk microbiome and should be regarded as hypothesis‐generating, as they may reflect compositional effects or indirect relationships rather than true biological interactions.

**Figure 6:**
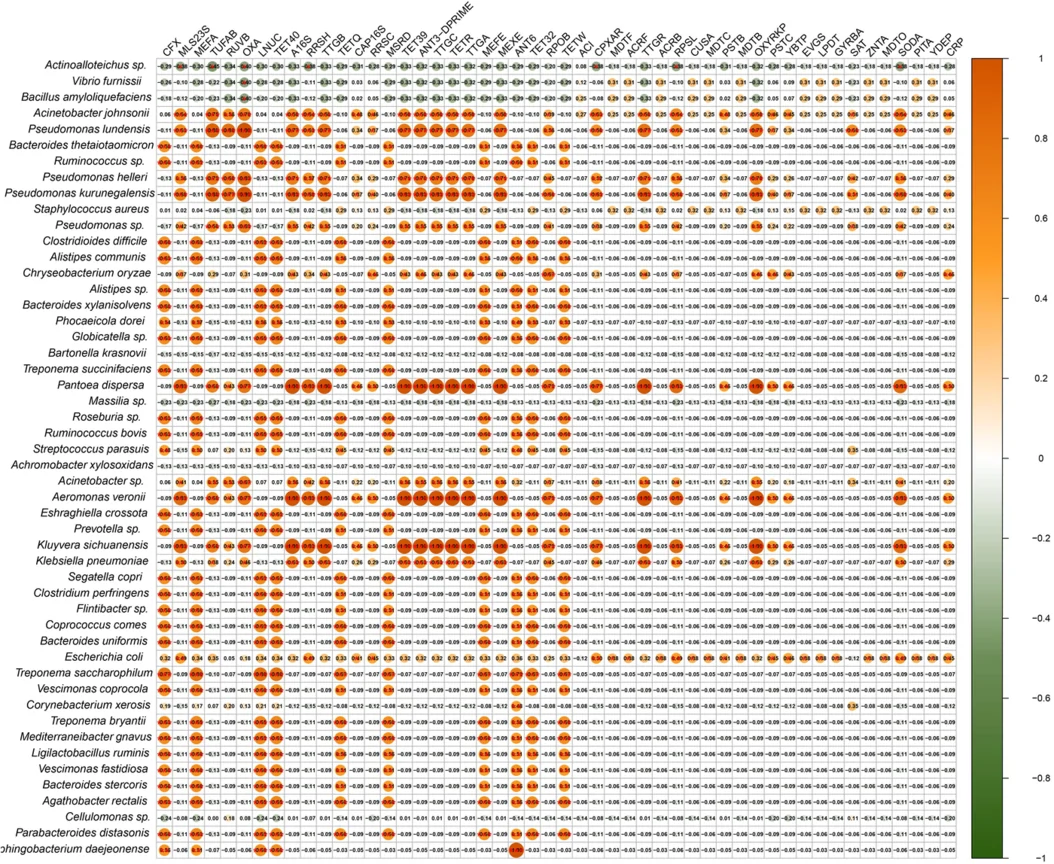
Spearman's correlation matrix between antibiotic resistance genes (ARGs) and the top abundant bacterial species. A correlation heatmap displays Spearman's rank coefficients between the relative abundance of the top 50 abundant bacterial species (*y*‐axis) and the abundance of various ARGs (*x*‐axis). Statistically significant correlations are represented by circles, with color (orange for positive, green for negative) and size scaled to the magnitude of the correlation coefficient. The color intensity and circle size correspond to the strength of the correlation, with larger and darker circles indicating stronger relationships. Statistical significance is denoted as follows: *p < 0.05, **p < 0.01, ***p < 0.001.

### 3.7 Correlation Between Functional Pathways and Top Abundant Bacterial Species

Spearman correlation analysis revealed significant associations (*p* < 0.05) between bacterial species and predicted KEGG functional pathways (Figure 7). *Actinoalloteichus* sp. and *V. furnissii* displayed consistent positive associations across membrane transport (*ρ* = 0.43–0.44), lipid metabolism (*ρ* = 0.44), and amino acid metabolism (*ρ* = 0.44–0.53), while showing negative correlations with endocrine and metabolic disease and sensory system pathways. *B. amyloliquefaciens* showed positive correlations with cellular community—eukaryotes (*ρ* = 0.25), lipid and immune metabolism pathways, and modest negative associations with neurodegenerative disease (*ρ* = −0.12) and endocrine system pathways. *A. johnsonii* showed strong positive associations with membrane transport (*ρ* = 0.53), amino acid metabolism (*ρ* = 0.53), and infectious disease: bacterial (*ρ* = 0.53), while exhibiting negative correlations with sensory system (*ρ* = −0.12) and endocrine and metabolic disease pathways. *P. lundensis* showed positive correlations with membrane transport (*ρ* = 0.42), lipid metabolism (*ρ* = 0.42), and xenobiotics biodegradation (*ρ* = 0.42), while correlating negatively with endocrine and metabolic disease (*ρ* = −0.10). *B. thetaiotaomicron* showed positive correlations with cellular community—eukaryotes (*ρ* = 0.26), carbohydrate metabolism (*ρ* = 0.25), replication and repair (*ρ* = 0.31), and immune disease (*ρ* = 0.04), with negative associations with circulatory system (*ρ* = −0.30) and endocrine and metabolic disease (*ρ* = −0.28). *Ruminococcus* sp. showed positive associations with carbohydrate metabolism (*ρ* = 0.25), replication and repair (*ρ* = 0.30), and biosynthesis of other secondary metabolites (*ρ* = 0.40), with negative correlations with circulatory system (*ρ* = −0.20) and sensory system pathways. *Pseudomonas* sp. similarly exhibited strong positive correlations with membrane transport (*ρ* = 0.54), lipid metabolism (*ρ* = 0.54), amino acid metabolism (*ρ* = 0.54), and xenobiotics biodegradation (*ρ* = 0.54), with negative associations with sensory system (*ρ* = −0.37) and endocrine and metabolic disease pathways. *P. helleri* and *P. kurunegalensis* both showed broad positive correlations with membrane transport (*ρ* = 0.48–0.54), lipid metabolism (*ρ* = 0.54–0.55), amino acid metabolism (*ρ* = 0.54–0.55), xenobiotics biodegradation (*ρ* = 0.54–0.55), and translation (*ρ* = 0.54–0.55), with negative associations with signaling molecules and interaction (*ρ* = −0.22 to −0.35) and sensory system pathways. *S. aureus* showed negative correlations with membrane transport (*ρ* = −0.14), lipid metabolism (*ρ* = −0.39), amino acid metabolism (*ρ* = −0.15), and xenobiotics biodegradation (*ρ* = −0.18), while showing positive associations with circulatory system (*ρ* = 0.15), nervous system (*ρ* = 0.15), and infectious disease: viral (*ρ* = 0.15) pathways. *C. difficile*, *A. communis*, *B. xylanisolvens*, *P. dorei*, and *Globicatella* sp. shared similar positive correlation profiles with carbohydrate metabolism (*ρ* ≈ 0.24–0.32), replication and repair (*ρ* ≈ 0.30–0.40), and biosynthesis of other secondary metabolites (*ρ* ≈ 0.30–0.45), with negative associations with circulatory system and endocrine and metabolic disease pathways. *B. krasnovii* showed notable negative correlations with membrane transport (*ρ* = −0.37), lipid metabolism (*ρ* = −0.43), amino acid metabolism (*ρ* = −0.43), and xenobiotics biodegradation (*ρ* = −0.43), while positively correlating with circulatory system (*ρ* = 0.06) and nervous system (*ρ* = 0.14) pathways. *T. succinifaciens*, *P. dispersa*, *Roseburia* sp., *R. bovis*, and *S. parasuis* showed positive correlations with carbohydrate and amino acid metabolism, replication and repair, and biosynthesis of secondary metabolites, alongside negative associations with circulatory system and endocrine and metabolic disease pathways. *A. xylosoxidans* exhibited broader negative correlations across membrane transport (*ρ* = −0.14), lipid metabolism (*ρ* = −0.35), amino acid metabolism (ρ = −0.33), and xenobiotics biodegradation (*ρ* = −0.88), with positive associations with circulatory system (*ρ* = 0.18) and nervous system pathways. *Acinetobacter* sp., *A. eromonas veronii*, *E. crossota*, *Prevotella* sp., *K. sichuanensis*, and *K. pneumoniae* broadly correlated positively with membrane transport (*ρ* = 0.16–0.48), amino acid metabolism (*ρ* = 0.32–0.53), lipid metabolism (*ρ* = 0.41–0.51), and infectious disease: bacterial (*ρ* = 0.32–0.53), with negative associations with endocrine system and sensory system pathways. *E. coli* exhibited positive correlations with signaling molecules and interaction (*ρ* = 0.32), infectious disease: viral (*ρ* = 0.26), nucleotide metabolism (*ρ* = 0.34), and folding, sorting and degradation (*ρ* = 0.35). *Massilia* sp. and *Chryseobacterium oryzae* showed divergent patterns, with *Massilia* sp. exhibiting strong negative correlations with most metabolic pathways (*ρ* = −0.30 to −0.34) and *C. oryzae* showing positive associations with lipid metabolism (*ρ* = 0.34) and translation (*ρ* = 0.39). The remaining taxa showed broadly similar positive correlations with carbohydrate metabolism, replication and repair, and biosynthesis of secondary metabolites, paired with consistent negative associations with circulatory system, endocrine system, and sensory system pathways. Overall, these associations describe co‐occurrence patterns between bacterial taxa and functional KEGG pathways and should be regarded as hypothesis‐generating, as they may reflect compositional effects or indirect relationships rather than true biological interactions (Figure 7).

**Figure 7:**
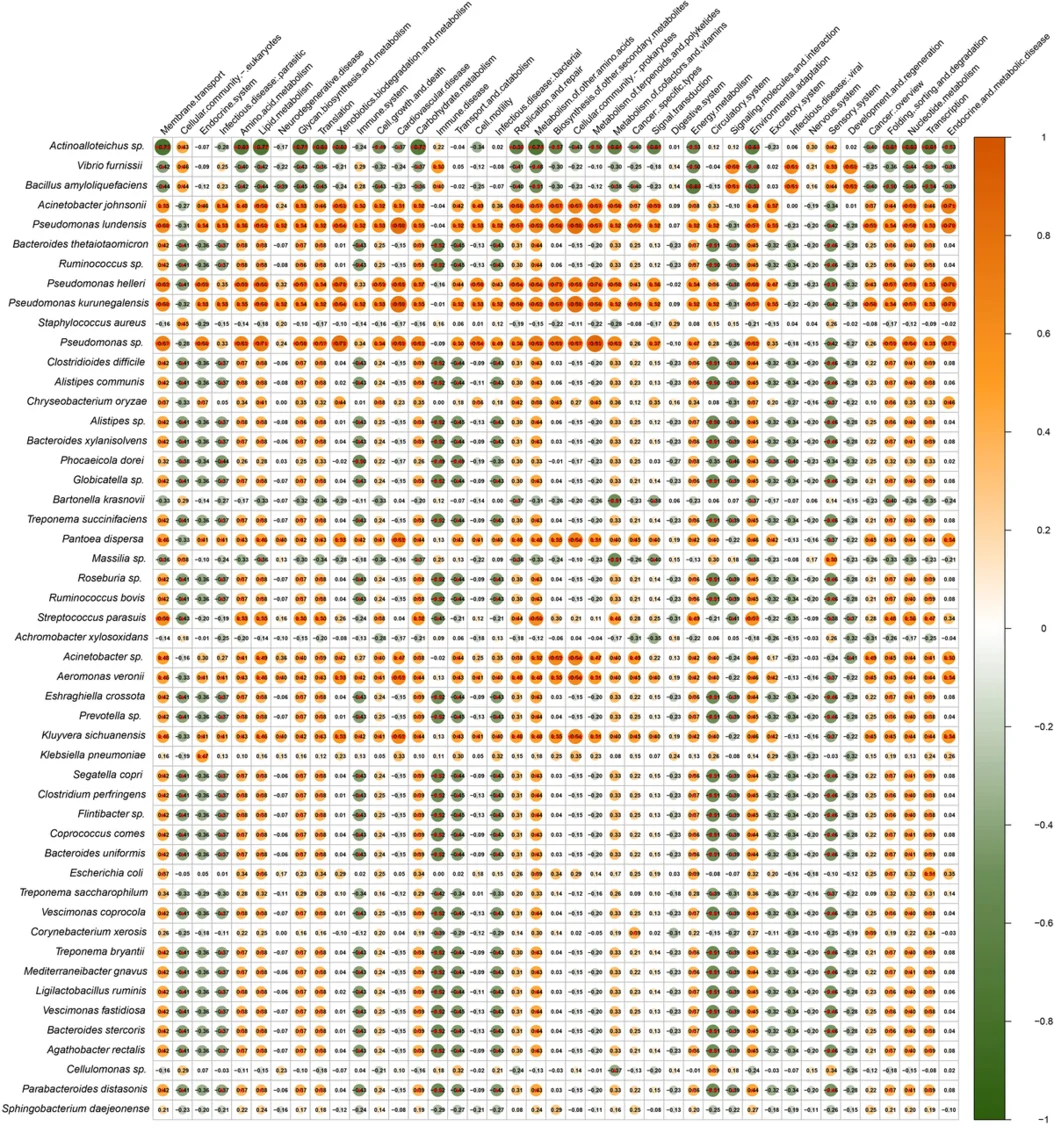
Spearman's correlation matrix between KEGG functional pathways and the top dominant bacterial species. A correlation heatmap displays Spearman's rank coefficients between the relative abundance of the top 50 most abundant bacterial species (*y*‐axis) and the abundance of various KEGG functional pathways (*x*‐axis). Statistically significant correlations are represented by circles, with color (orange for positive, green for negative) and size scaled to the magnitude of the correlation coefficient. The color intensity and circle size correspond to the strength of the correlation, with larger and darker circles indicating stronger relationships. Statistical significance is denoted as follows: *p < 0.05, **p < 0.01, ***p < 0.001.

## 4 Discussion

### 4.1 Milk Bacteriome Dysbiosis Across Health Status and Treatment Groups

This study provides comprehensive evidence of significant dysbiosis in the milk bacteriome of cows with SCM and following antibiotic treatment, characterized by reduced alpha diversity, pronounced shifts in microbial community composition, and substantial enrichment of ARGs and metabolic functions. These findings align with a growing body of literature documenting mastitis‐associated microbiome disruption and resistome expansion in dairy cattle (Alessandri et al. 2023; Derakhshani et al. 2020; Hoque et al. 2020, 2019; Salman et al. 2023b). Despite significantly higher microbial richness and diversity in HM samples compared to SCM and ANT groups, overall community composition (beta diversity) remained statistically indistinguishable (PERMANOVA, *p* = 0.142). This suggests that while diversity indices change, the core compositional shift might be subtler or more driven by specific taxa rather than a complete restructuring of the microbial ecosystem. The observed decline in microbial diversity in both SCM and ANT samples corroborates established evidence of bacteriome dysbiosis induced by intramammary infections and antimicrobial interventions (Hoque and Rahman 2026; Pang et al. 2018; Steinberg et al. 2022).

At the taxonomic level, the clear enrichment of the phylum Pseudomonadota in ANT (51.75%) and SCM (37.80%) samples is clinically concerning, as this phylum encompasses opportunistic pathogens including *E. coli*, *K. pneumoniae*, and *A. johnsonii*—well‐recognized mastitis agents (Hoque et al. 2020, 2022a; Polveiro et al. 2022). The persistence of Pseudomonadota in ANT samples suggests that conventional antibiotic therapy may not effectively suppress these taxa, potentially due to biofilm formation, acquired resistance mechanisms, or the presence of persister cells (Cheng et al. 2019; Hoque et al. 2020). At the species level, the striking enrichment of *S. aureus* (1.74%) in the SCM group confirms its classic etiological role in subclinical intramammary infections (Bhatt et al. 2012; Park et al. 2022). Conversely, antibiotic treatment selected heavily for *A. johnsonii*, which peaked in the ANT group (10.52%) but was nearly absent in SCM (< 0.01%), suggesting that the active mastitic microenvironment is unfavorable to its survival until antibiotic pressure suppresses competing taxa (Hassan et al. 2023; Hoque et al. 2018, 2024). Importantly, health‐associated commensals such as *P. lundensis*, *B. thetaiotaomicron*, and *L. lactis* were heavily depleted or entirely absent in SCM and ANT samples, confirming that disease‐associated dysbiosis involves the severe displacement of beneficial commensals that are vital for maintaining domestic udder homeostasis (Newbury et al. 2022; Rahman et al. 2025; Rahman et al. 2024; Rahman et al. 2024; Sora et al. 2022). However, as ANT milk samples were collected only 24 h after the final antibiotic dose (Day 8), residual antimicrobial activity may have continued to influence the milk microbiota and resistome. Thus, the observed profiles may primarily reflect the immediate effects of antibiotic exposure rather than stable or persistent ecological changes following treatment cessation.

### 4.2 Influence of Host Breed and Age on Compositional Successions

Our results demonstrate that host intrinsic factors, specifically breed type and age, play a significant role in modulating the milk microbiota. Among the evaluated breeds, LZ cows displayed the highest microbial richness and Shannon diversity compared to HFC and SC cows. These quantitative variations likely reflect differences in innate immune baselines, genetic adaptations, or breed‐specific management practices (Cremonesi et al. 2018; Salman et al. 2023b; Steinberg et al. 2022). This breed‐specific pattern was further reflected at the species level; for example, *A. johnsonii* was enriched in LZ cows (6.35%), while *P. lundensis* showed a striking, highly specific enrichment in SC cows (7.12%) compared to negligible levels (< 0.80%) in HFC and LZ. These observations suggest that host genetics may establish distinct microenvironmental niches within the mammary gland that favor or suppress specific bacterial lineages (Campos et al. 2022; Hoque et al. 2020; Mariadassou et al. 2023).

Age‐associated trends revealed that microbial diversity peaks in Middle‐aged animals (5–8 years) before declining in older groups. This confirms that the bovine milk microbiome undergoes ongoing, non‐linear successional shifts across the host's productive lifespan (Duarte et al. 2025). Our study captured a clear progressive succession of the dominant core taxa; *Actinoalloteichus* sp., *V. furnissii*, and *B. amyloliquefaciens* all exhibited steady, linear increases from Young to Older cows. In contrast, beneficial or transient species like *P. lundensis* and *A. johnsonii* peaked in Young animals and experienced sharp declines or complete elimination with advancing age. Furthermore, the observation that key pathogens like *S. aureus* and *C. difficile* exhibited peak abundances specifically in Middle‐aged cows suggests a period of heightened susceptibility to microbial dysbiosis during the peak lactation years, whereas older cows become increasingly dominated by highly persistent, specialized commensal consortia. These findings align with a growing body of literature documenting age‐associated microbial succession in the bovine mammary gland and its influence on udder health and disease susceptibility (Addis et al. 2016; Derakhshani et al. 2018; G. Zhang et al. 2019). Longitudinal studies have demonstrated that lactation stage and parity significantly modulate milk microbiome composition, with distinct taxonomic shifts occurring as cows progress through their productive lifespan (Zhu et al. 2023). The progressive enrichment of Actinomycetota and concurrent decline of Pseudomonadota with advancing age observed in our study corroborates previous reports of age‐dependent phylum‐level shifts in bovine milk (Taponen et al. 2017). Moreover, the increased colonization by persistent commensal consortia in older cows may reflect immune system maturation and ecological niche consolidation, potentially contributing to reduced susceptibility to opportunistic infections (Oikonomou et al. 2020; Rainard et al. 2021). The age‐specific enrichment of mastitis‐associated pathogens during middle age aligns with epidemiological evidence indicating higher clinical mastitis incidence during peak lactation periods, particularly in high‐yielding dairy cows (Barkema et al. 2006; De Vliegher et al. 2012). Collectively, these age‐dependent dynamics underscore the importance of considering physiological age as a critical determinant of mammary gland microbial ecology and highlight the need for age‐specific management strategies to mitigate mastitis risk throughout the dairy cow's productive life.

### 4.3 Cooperative Ecological Networks in the Milk Ecosystem

A defining feature of the bovine milk bacteriome highlighted by our network analysis is its highly skewed structure, where over 70% of the entire community in the treated ecosystem is dominated by just two species: *Actinoalloteichus* sp. and *V. furnissii*. Crucially, all pairwise Spearman correlations within the network data set were positive, indicating that the relationships among the resident taxa are predominantly cooperative or co‐occurring rather than competitive. This lack of negative or antagonistic links suggests that the milk microbiome operates via syntrophic, mutually supportive microbial consortia (Hoque et al. 2022a; C. Ma et al. 2018). Among these, the near‐perfect positive correlation between *V. furnissii* and *B. amyloliquefaciens* represents a powerful inter‐species co‐occurrence hub. Similarly, *A. johnsonii* demonstrated strong ecological coupling with multiple *Pseudomonas* species (including *P. helleri*, *P. kurunegalensis*, and *P. lundensis*), pointing to a robust, co‐selected Gram‐negative bacterial complex. Furthermore, our analysis identified a tightly interconnected commensal consortium composed entirely of gut‐associated genera (including *B. thetaiotaomicron*, *B. xylanisolvens*, *Alistipes* sp., *C. difficile*, and *T. succinifaciens*) that displayed near‐perfect mutual co‐occurrence. This highly synchronized network likely represents a conserved internal entero‐mammary pathway or a shared environmental source inoculating the milk ecosystem, where these taxa co‐exist syntrophically without competitive exclusion (Newbury et al. 2022; Rahman et al. 2025, 2024; Sora et al. 2022).

### 4.4 Resistome Dynamics and Therapeutic Selection Pressures

The metagenomic resistome profiling conducted in this study provides clear evidence of how subclinical infection and subsequent therapy reshape the ARG landscape. SCM microbiomes possessed the largest overall fraction and diversity of ARGs (161 out of 194 total ARGs; 68.04% unique), cementing the unmanaged mastitic udder as a massive natural reservoir for diverse resistance determinants. AMR in mastitis pathogens represents a growing concern driven by excessive antibiotic use in dairy farming (Chowdhury et al. 2025). The substantial ARG burden in SCM likely reflects microbial dysbiosis creating conducive environments for resistance determinant expansion (Vasco et al. 2023). Conversely, the reduced ARG diversity in ANT samples may reflect the selective elimination of susceptible taxa and dominance of a few resistant strains (Abegewi et al. 2022; Amarlapudi et al. 2025; Z. Ma et al. 2021). The significant enrichment of macrolide resistance genes (*MLS23S*), aminoglycoside resistance determinants (*A16S*, *RRSH*), and β‐lactamase genes (*OXA*) in ANT samples directly reflects therapeutic selection pressures from the ampicillin‐azithromycin regimen administered (Winther et al. 2024). The co‐occurrence of these genes with efflux pump genes (*TTGB*, *TTGC*) in ANT samples suggests emerging cross‐resistance mechanisms (Novelli and Bolla 2024). The strong positive correlation observed between macrolide and aminoglycoside resistance genes in ANT milk strongly supports the occurrence of treatment‐driven co‐selection, where exposing the microbiome to a specific drug simultaneously selects for linked resistance mechanisms (Wang et al. 2022; Z. Zhang et al. 2022). It is also noteworthy that while HM samples contained the lowest ARG burden, they still maintained background reservoirs of resistance factors carried by commensals, such as tetracycline (*TETQ*) and elfamycin (*TUFAB*) resistance, emphasizing that the baseline milk microbiome naturally harbors low‐level resistome determinants even in the absence of disease or therapeutic intervention (Patangia et al. 2023; Sahoo et al. 2024).

### 4.5 Taxon‐ARG Co‐Occurrence Hubs

Our correlation analysis identified key bacterial taxa that serve as major reservoirs or hubs for specific ARGs within the milk metagenome. *E. coli* emerged as a prominent multi‐resistance hub, displaying strong, uniform positive associations (*r* = 0.63) with tetracycline resistance genes (*TET32*, *TETQ*, *TETM*), aminoglycoside resistance determinants (*ANT3‐DPRIME*), and multidrug efflux pump systems (*TTGB*, *TTGC*). This multi‐drug resistant profile highlights *E. coli* as a critical target for antimicrobial stewardship in dairy herds (Hoque et al. 2016, 2024; Siddique et al. 2025). Similarly, *A. johnsonii* was strongly coupled with aminoglycoside resistance (*A16S*, *r* = 0.79) and beta‐lactamase genes (*CFX*, *r* = 0.66), which explains its competitive dominance and enrichment in the ANT milk samples following therapeutic exposure (Hoque et al. 2020, 2022a). The opportunistic pathogen *K. pneumoniae* similarly acted as a resistance hub for efflux systems and tetracycline resistance (Hoque et al. 2019). Furthermore, the community‐dominating taxa *Actinoalloteichus* sp. and *V. furnissii* maintained broad, baseline positive correlations across nearly all major ARG classes, including beta‐lactams, MLS, and tetracyclines (*r* = 0.31–0.34). This baseline resistance capacity likely underpins their ability to remain completely unsuppressed and dominant during active antibiotic treatment (Alessandri et al. 2023). However, the associations between bacterial taxa and ARGs observed in this study represent co‐occurrence patterns derived from correlation‐based analysis of compositional data, and should not be interpreted as direct ecological interactions or gene transfer events. These findings are therefore hypothesis‐generating and require validation using host‐resolved and functional approaches in larger, multi‐farm and longitudinal studies.

### 4.6 Metabolic Functional Pathway (KEGG) Alterations

The predicted functional variations across the milk metagenome further clarify the adaptive mechanisms employed by the bacteriome during dysbiosis. *Acinetobacter* and *Pseudomonas* species (including *A. johnsonii*, *P. lundensis*, *P. helleri*, and *P. kurunegalensis*) displayed powerful, consistent positive correlations with core metabolic pathways, specifically membrane transport, amino acid metabolism, lipid metabolism, and xenobiotic biodegradation (*r* = 0.42–0.55). This metabolic versatility is highly indicative of enhanced stress tolerance; by upregulating membrane transport and lipid/xenobiotic catabolism, these Gram‐negative taxa can actively detoxify their immediate microenvironment and drive cellular efflux, facilitating survival and competitive dominance in antibiotic‐exposed settings (Ayemele et al. 2021; El‐Karim et al. 2023; Steinberg et al. 2022). In sharp contrast, the primary pathogen *S. aureus* exhibited significant negative correlations with these core metabolic and biodegradation pathways, while correlating positively with host‐interactive processes such as circulatory systems and viral infectious diseases. This suggests that while *S. aureus* relies on specialized virulence factors rather than broad metabolic adaptability (Hoque et al. 2018), opportunistic Gram‐negative bacteria leverage extensive metabolic rewiring to thrive during inflammation and treatment. Lastly, the tightly coupled gut‐associated commensal consortium demonstrated uniform positive links with carbohydrate metabolism, replication and repair, and the biosynthesis of secondary metabolites (Hu et al. 2022; Zhao et al. 2022), reflecting a classic, stable homeostatic lifestyle focused on energy harvesting and cellular maintenance rather than stress‐induced detoxification.

### 4.7 Limitations and Future Directions

While our shotgun metagenomic analysis provides valuable, high‐resolution insights into the milk bacteriome, resistome, and functional landscapes associated with bovine mastitis, several key limitations must be addressed. First, samples were collected from a single commercial farm with relatively small group sizes (*n* = 10 per condition), which may introduce farm‐specific environmental biases and reduce the statistical power needed to capture rare taxa or generalizable global ecological shifts. Second, all treated animals received a combined ampicillin–azithromycin regimen, reflecting clinical practice but preventing assessment of the independent effects of each antimicrobial on the microbiome and resistome. As these drugs belong to different antimicrobial classes, they may exert distinct selective pressures. Third, ANT samples were collected only 24 h after the final antibiotic dose; therefore, residual antimicrobial activity may have influenced the observed microbial and resistome profiles, potentially reflecting acute drug exposure rather than persistent post‐treatment ecological changes. Additional sampling beyond the withdrawal period would help distinguish transient antibiotic effects from longer‐term resistome alterations. Fourth, shotgun metagenomics detects microbial DNA and functional genes but cannot reliably distinguish viable from non‐viable microorganisms or determine whether detected ARGs and metabolic pathways are actively expressed. Fifth, the study had a mixed design, with SCM and ANT samples paired within 10 cows and HM samples from independent untreated cows. Cow‐level pairing was accounted for in the SCM–ANT PERMANOVA, although the small sample size may have limited statistical power. Furthermore, all reported associations between bacterial species, ARGs, and KEGG pathways are based on correlation patterns derived from compositional data; they represent co‐occurrence trends and must not be interpreted as definitive proof of direct biological or mechanistic interactions. Future longitudinal, multi‐farm studies integrating milk somatic cell count, culture‐based confirmation, metatranscriptomics, and metabolomics are needed to improve diagnostic accuracy, validate these findings, and clarify the mechanisms linking microbiome, resistome, and metabolome dynamics in bovine mastitis.

## 5 Conclusion

This study demonstrates that subclinical mastitis and antibiotic treatment substantially reshape the milk bacteriome, resistome, and metabolic functional landscape of dairy cows. Shotgun metagenomic analysis revealed a marked reduction in microbial diversity in SCM and ANT milk compared with healthy milk, accompanied by distinct shifts in bacterial community composition. Healthy milk maintained the highest microbial richness, particularly in Local Zebu and Middle‐aged (5–8 years) cows. SCM milk was characterized by the enrichment of mastitis‐associated taxa, including *S. aureus*, along with dominance of *Actinoalloteichus* sp. and *V. furnissii*, whereas ANT milk showed increased *A. johnsonii* and HM harbored beneficial commensals including *P. lundensis* and *L. lactis*. Breed‐specific patterns revealed *P. lundensis* enrichment in SC, while age‐related shifts showed a progressive increase of *Actinoalloteichus* sp. from Young (33.63%) to Older cows (39.20%). SCM milk harbored the largest repertoire of ARGs, highlighting its role as an important reservoir of resistance determinants. In contrast, ANT milk showed substantial enrichment of macrolide‐, aminoglycoside‐, and β‐lactam‐resistance genes, indicating strong treatment‐driven selection pressure. Correlation analyses linked *E. coli* and *A. johnsonii* with multiple ARGs. Functional profiling further suggested that dominant taxa, particularly *Acinetobacter* and *Pseudomonas* species, are closely associated with membrane transport, lipid metabolism, amino acid metabolism, and xenobiotic biodegradation pathways, reflecting enhanced metabolic adaptability under disease and antibiotic exposure. Collectively, these findings provide comprehensive evidence that both SCM and antimicrobial intervention disrupt the ecological balance of the milk bacteriome and promote resistance enrichment, with breed and age serving as additional modulatory factors. The study underscores the need for routine AMR monitoring and judicious antibiotic use to curb resistance within the dairy sector of Bangladesh and comparable dairy systems.

## Author Contributions

**M. Nazmul Hoque:** conceptualization, investigation, funding acquisition, writing – original draft, methodology, validation, visualization, writing – review and editing, formal analysis, project administration, data curation, resources. **M. Shaminur Rahman:** conceptualization, investigation, writing – original draft, methodology, visualization, writing – review and editing, formal analysis, software, data curation. **Md. Golzar Hossain:** writing – original draft, methodology, validation, software, data curation. **Md. Tanvir Rahman:** investigation, writing – review and editing, supervision, validation. **Tofazzal Islam:** conceptualization, investigation, writing – review and editing, validation, supervision, resources. **M. Anwar Hossain:** validation; writing – review and editing, project administration, supervision, resources.

## Ethics Statement

The authors have nothing to report.

## Consent

The authors have nothing to report.

## Conflicts of Interest

The authors declare no conflicts of interest.

## Supporting information


**Figure S1:** Phylum level taxonomic composition of bacteriomes. Venn diagrams display the composition of bacterial communities at phylum level according to sample types (SCM, ANT, and HM), (B) breeds (HFC, LZ, and SC), and (C) age (Young, Middle, Older) of the dairy cows. Shared phyla are highlighted in yellow circle.

**Figure S2:** Species level taxonomic composition of bacteriomes. Venn diagrams display the composition of bacterial communities at the species level according to sample types (SCM, ANT and HM), (B) breeds (HFC, LZ and SC), and (C) age (Young, Middle, Older) of the dairy cows. Shared species are highlighted in yellow circle.

**Figure S3:** Antimicrobial resistance genes (ARGs) repertoire in the subclinical mastitis milk (SCM), antibiotic treated milk (ANT), and healthy milk (HM) samples. Shared ARG is highlighted in yellow circle.

Supporting File:

**Table S1:** Study sample information, number of reads (before and after trimming) and SRA accession numbers of the whole metagenome sequences.

**Data S1:** Taxonomic information of the milk bacteriomes (phyla to species/strain levels) identified in different sample groups in dairy cows in Bangladesh.

## Data Availability

The shotgun whole metagenome sequencing data are available at the NCBI Sequence Read Archive (SRA) under BioProject accession number PRJNA1081607. The accession numbers for all 30 SRA experiments are listed in Table S1.
