# Supplementary material for: Subclinical Mastitis and Antibiotic Exposure Reshape the Milk Bacteriome, Resistome, and Metabolic Functional Profiles in Dairy Cows
Source: Microbiologyopen. 2026 Oct 12;15(5):e70440. doi: 10.1002/mbo3.70440 (PMC13657076; doi:10.1002/mbo3.70440)
Supplement: Supplementary file 1 [file MBO3-15-e70440-s002.docx]

**Supplementary Figures**

**Figure S1.** Phylum level taxonomic composition of bacteriomes. Venn diagrams display the composition of bacterial communities at phylum level according to sample types (SCM, ANT and HM), (B) breeds (HFC, LZ and SC), and (C) age (Young, Middle, Older) of the dairy cows. Shared phyla are highlighted in yellow circle.

**Figure S2.** Species level taxonomic composition of bacteriomes. Venn diagrams display the composition of bacterial communities at species level according to sample types (SCM, ANT and HM), (B) breeds (HFC, LZ and SC), and (C) age (Young, Middle, Older) of the dairy cows. Shared species are highlighted in yellow circle.

**Figure S3.** Antimicrobial resistance genes (ARGs) repertoire in the subclinical mastitis milk (SCM), antibiotic treated milk (ANT), and healthy milk (HM) samples. Shared ARG is highlighted in yellow circle.
