# Supplementary material for: Subclinical Mastitis and Antibiotic Exposure Reshape the Milk Bacteriome, Resistome, and Metabolic Functional Profiles in Dairy Cows
Source: Microbiologyopen. 2026 Oct 12;15(5):e70440. doi: 10.1002/mbo3.70440 (PMC13657076; doi:10.1002/mbo3.70440)
Supplement: Supplementary file 2 [file MBO3-15-e70440-s004.docx]

**Table S1.** Study sample information, number of reads (before and after trimming) and SRA accession numbers of the whole metagenome sequences.

| **Sl. No.** | **Sample ID** | **Sample Type** | **Breed** | **Age (Years)** | **Lactation Stage (Days after parturition)** | **Milk Yield (Litre)** | **Farm Location** | **No. of raw reads (before trimming)** | **No. of quality reads (after trimming)** | **No. of mapped reads** | **SRA accessions** |
| --- | --- | --- | --- | --- | --- | --- | --- | --- | --- | --- | --- |
| 1 | SCM1 | SCM | HFC | 7 | 45 | 22 | Dhaka | 12113746 | 12060664 | 1875533 | [SRR28341692](https://www.ncbi.nlm.nih.gov/sra/SRX23948217%5baccn%5d) |
| 2 | SCM2 | SCM | HFC | 4.5 | 22 | 14 | Dhaka | 11843987 | 11776824 | 1081465 | [SRR28341686](https://www.ncbi.nlm.nih.gov/sra/SRX23948223%5baccn%5d) |
| 3 | SCM3 | SCM | SC | 5 | 7 | 7 | Dhaka | 15008310 | 14656341 | 1484361 | [SRR28341675](https://www.ncbi.nlm.nih.gov/sra/SRX23948234%5baccn%5d) |
| 4 | SCM4 | SCM | LZ | 6 | 60 | 3 | Dhaka | 12070051 | 12034338 | 559680 | [SRR28341664](https://www.ncbi.nlm.nih.gov/sra/SRX23948245%5baccn%5d) |
| 5 | SCM5 | SCM | LZ | 5 | 40 | 4 | Dhaka | 11999831 | 11953257 | 383352 | [SRR28341663](https://www.ncbi.nlm.nih.gov/sra/SRX23948246%5baccn%5d) |
| 6 | SCM6 | SCM | SC | 8 | 15 | 6 | Dhaka | 8712546 | 8489918 | 278135 | [SRR28341691](https://www.ncbi.nlm.nih.gov/sra/SRX23948218%5baccn%5d) |
| 7 | SCM7 | SCM | LZ | 6 | 30 | 5 | Dhaka | 15008310 | 14656341 | 308310 | [SRR28341690](https://www.ncbi.nlm.nih.gov/sra/SRX23948219%5baccn%5d) |
| 8 | SCM8 | SCM | HFC | 5 | 7 | 18 | Dhaka | 11920062 | 11896202 | 91360 | [SRR28341689](https://www.ncbi.nlm.nih.gov/sra/SRX23948220%5baccn%5d) |
| 9 | SCM9 | SCM | SC | 10 | 10 | 12 | Dhaka | 12887239 | 12642929 | 1178180 | [SRR28341688](https://www.ncbi.nlm.nih.gov/sra/SRX23948221%5baccn%5d) |
| 10 | SCM10 | SCM | HFC | 8 | 15 | 18 | Dhaka | 19480895 | 19272605 | 251571 | [SRR28341687](https://www.ncbi.nlm.nih.gov/sra/SRX23948222%5baccn%5d) |
| 11 | ANT1 | ANT | HFC | 7 | 45 | 22 | Dhaka | 8215147 | 7910230 | 308752 | [SRR28341685](https://www.ncbi.nlm.nih.gov/sra/SRX23948224%5baccn%5d) |
| 12 | ANT2 | ANT | HFC | 4.5 | 22 | 14 | Dhaka | 7122952 | 6854102 | 115384 | [SRR28341684](https://www.ncbi.nlm.nih.gov/sra/SRX23948225%5baccn%5d) |
| 13 | ANT3 | ANT | SC | 5 | 7 | 7 | Dhaka | 8473979 | 8254757 | 291453 | [SRR28341683](https://www.ncbi.nlm.nih.gov/sra/SRX23948226%5baccn%5d) |
| 14 | ANT4 | ANT | LZ | 6 | 60 | 3 | Dhaka | 10910737 | 9753576 | 628299 | [SRR28341681](https://www.ncbi.nlm.nih.gov/sra/SRX23948228%5baccn%5d) |
| 15 | ANT5 | ANT | LZ | 5 | 40 | 4 | Dhaka | 12130996 | 12076039 | 142524 | [SRR28341682](https://www.ncbi.nlm.nih.gov/sra/SRX23948227%5baccn%5d) |
| 16 | ANT6 | ANT | SC | 8 | 15 | 6 | Dhaka | 8015147 | 7810230 | 221500 | [SRR28341680](https://www.ncbi.nlm.nih.gov/sra/SRX23948229%5baccn%5d) |
| 17 | ANT7 | ANT | LZ | 6 | 30 | 5 | Dhaka | 7358494 | 7204336 | 1047845 | [SRR28341679](https://www.ncbi.nlm.nih.gov/sra/SRX23948230%5baccn%5d) |
| 18 | ANT8 | ANT | HFC | 5 | 7 | 18 | Dhaka | 8031995 | 7829594 | 909767 | [SRR28341678](https://www.ncbi.nlm.nih.gov/sra/SRX23948231%5baccn%5d) |
| 19 | ANT9 | ANT | SC | 10 | 10 | 12 | Dhaka | 11118015 | 10339228 | 177002 | [SRR28341677](https://www.ncbi.nlm.nih.gov/sra/SRX23948232%5baccn%5d) |
| 20 | ANT10 | ANT | HFC | 8 | 15 | 18 | Dhaka | 7799937 | 7572239 | 107620 | [SRR28341676](https://www.ncbi.nlm.nih.gov/sra/SRX23948233%5baccn%5d) |
| 21 | HM1 | HM | HFC | 5.5 | 22 | 14 | Dhaka | 12096858 | 12066530 | 71227 | [SRR28341674](https://www.ncbi.nlm.nih.gov/sra/SRX23948235%5baccn%5d) |
| 22 | HM2 | HM | HFC | 6 | 10 | 9 | Dhaka | 10097985 | 8884528 | 102098 | [SRR28341673](https://www.ncbi.nlm.nih.gov/sra/SRX23948236%5baccn%5d) |
| 23 | HM3 | HM | HFC | 7 | 35 | 12 | Dhaka | 3301658 | 3249161 | 59822 | [SRR28341672](https://www.ncbi.nlm.nih.gov/sra/SRX23948237%5baccn%5d) |
| 24 | HM4 | HM | HFC | 4 | 5 | 19 | Dhaka | 12259145 | 12221271 | 349108 | [SRR28341670](https://www.ncbi.nlm.nih.gov/sra/SRX23948239%5baccn%5d) |
| 25 | HM5 | HM | SC | 6.5 | 15 | 10 | Dhaka | 11018572 | 10071382 | 50954 | [SRR28341671](https://www.ncbi.nlm.nih.gov/sra/SRX23948238%5baccn%5d) |
| 26 | HM6 | HM | SC | 4 | 5 | 8 | Dhaka | 9928322 | 9706011 | 470371 | [SRR28341669](https://www.ncbi.nlm.nih.gov/sra/SRX23948240%5baccn%5d) |
| 27 | HM7 | HM | SC | 7 | 45 | 12 | Dhaka | 8480389 | 8260368 | 15713 | [SRR28341668](https://www.ncbi.nlm.nih.gov/sra/SRX23948241%5baccn%5d) |
| 28 | HM8 | HM | LZ | 7 | 55 | 5 | Dhaka | 17227741 | 17037343 | 17364 | [SRR28341667](https://www.ncbi.nlm.nih.gov/sra/SRX23948242%5baccn%5d) |
| 29 | HM9 | HM | LZ | 10 | 40 | 4 | Dhaka | 10373218 | 10119570 | 49263 | [SRR28341666](https://www.ncbi.nlm.nih.gov/sra/SRX23948243%5baccn%5d) |
| 30 | HM10 | HM | LZ | 8.5 | 24 | 4 | Dhaka | 11988452 | 11960796 | 21577 | [SRR28341665](https://www.ncbi.nlm.nih.gov/sra/SRX23948244%5baccn%5d) |

SCM: Subclinical Mastitis Milk; ANT: Antibiotics treated Milk; HM: Healthy Cows Milk; HFC: Holstein Friesian Cross; SC: Sahiwal Cross; and LZ: Local Zebu, Age Group: Young = < 5 years, Middle-Aged = 5.0-8.0 years, Older = > 8.0 years.
